# A Low-Cost Modular Platform for Heterogeneous Data Acquisition with Accurate Interchannel Synchronization

**DOI:** 10.3390/s151027374

**Published:** 2015-10-27

**Authors:** José Luis Blanco-Claraco, Javier López-Martínez, José Luis Torres-Moreno, Antonio Giménez-Fernández

**Affiliations:** Engineering Department, University of Almería, Ctra. de Sacramento s/n, La Cañada 04120, Spain; E-Mails: jlm167@ual.es (J.L.-M.); jltmoreno@ual.es (J.L.T.-M.); agimfer@ual.es (A.G.-F.)

**Keywords:** accelerometers, impact tests, firmware design, microcontroller, open source, quadrature encoders, vibration analysis

## Abstract

Most experimental fields of science and engineering require the use of data acquisition systems (DAQ), devices in charge of sampling and converting electrical signals into digital data and, typically, performing all of the required signal preconditioning. Since commercial DAQ systems are normally focused on specific types of sensors and actuators, systems engineers may need to employ mutually-incompatible hardware from different manufacturers in applications demanding heterogeneous inputs and outputs, such as small-signal analog inputs, differential quadrature rotatory encoders or variable current outputs. A common undesirable side effect of heterogeneous DAQ hardware is the lack of an accurate synchronization between samples captured by each device. To solve such a problem with low-cost hardware, we present a novel modular DAQ architecture comprising a base board and a set of interchangeable modules. Our main design goal is the ability to sample all sources at predictable, fixed sampling frequencies, with a reduced synchronization mismatch (<1 μs) between heterogeneous signal sources. We present experiments in the field of mechanical engineering, illustrating vibration spectrum analyses from piezoelectric accelerometers and, as a novelty in these kinds of experiments, the spectrum of quadrature encoder signals. Part of the design and software will be publicly released online.

## 1. Introduction

Data acquisition systems (DAQs) are the specialized pieces of hardware and software in charge of sampling real-world electrical signals and converting them into numerical values suitable for processing in digital computers. Most fields of science and engineering today require, at some point, the acquisition of analog or digital signals from different sensors, hence the transversal nature of DAQ applications. However, the specific requisites demanded of a DAQ system depend heavily on the specific experiment at hand, which explains the large number of different, mutually-incompatible commercial solutions. First of all, we could classify DAQs as [1]: (i) computer-based systems, where the electronic boards can be connected to an existing computer via local buses, such as USB or PCI; (ii) instruments, such as oscilloscopes or spectrum analyzers, which may be connected to a computer or can operate autonomously; and (iii) specialized modular designs, such as National Instruments’ solution PXI [2], where a computer-based controller acts as a host for interchangeable modules for specialized inputs or outputs. Each design has its own pros and cons. For example, Type (i) DAQs are typically more inexpensive than the rest, at the cost of not offering the most advanced features. In turn, it is common to find that the most stringent specifications are only achieved by Type (ii) DAQs, with a much higher cost. Finally, Type (iii) systems tend to offer a trade-off between the other two types.

The DAQ proposed in this work belongs to the latter group; it is the first low-cost, modular DAQ proposed in the literature. Our modular DAQ architecture comprises a base board with four slots in which a set of interchangeable modules can be inserted (refer to Figure 2), each one specifically designed for a particular kind of sensor or actuator. In the present work, modules will be described for two kinds of signals: analog voltage inputs and incremental quadrature encoder inputs. In our proposed platform, the number of inputs of each kind available in a DAQ can be increased by inserting multiple identical modules into the expansion slots. Before describing the proposal in detail, we will briefly review some previous related works found in the technical literature.

Regarding the specific target application of our system, which is performing multichannel vibration and impact tests in mechanical engineering, many other previous works describe DAQs for inertial units based on Microelectromechanical Systems (MEMS) technology, piezoelectric accelerometers and incremental quadrature encoders. For example, fatigue-induced crack diagnostics in gears is studied in [3] by means of quadrature encoders read through off-the-shelf National Instruments DAQ boards. Only one sensor type is employed in those tests, hence the lack of discussion about the synchronization of different sensor types. In [4], two biaxial MEMS accelerometers (ADXL210) are sampled with a commercial DAQ (Biomedical Monitoring BM42) at 1 kS/s, apparently without simultaneous sampling of all channels.

Some custom DAQ designs with low cost as the main priority have been also proposed. For example, [5] presents a board based on a Microchip eight-bit microcontroller (PIC18F27J53) running at 12 MHz as a single-chip board, capable of 12-bit multiplexed analog input sampling. A similar design can be found in [6], although an additional 22-bit Delta-Sigma Analog-To-Digital (ADC) chip (MCP3553) was incorporated for small signal acquisition. Another, much more elaborate proposal is Open DAQ [1], designed with low cost as the primary goal and providing eight multiplexed analog inputs and one analog output. The board is built upon an Atmel AVR ATMEGA644 microcontroller, sufficient for relatively high sample rates (100 kS/s) in simple systems with only one ADC chip. Regarding DAQs suitable for distributed signal acquisition, fundamentally different approaches can be followed depending on the size of the area to cover and on whether the priorities are reliability, cost or synchronization accuracy. Despite using quartz crystals and Phase-Locked-Loops (PLLs) for clock generation, in practice, two computers will never run their internal clocks at exactly the same rate since clocks from different motherboards often have relatively large drifts. Example reports of realistic values for this drift are around 10 μs/s in [7] and between 6 μS/s and 83 μS/s in [8]. Hence the fundamental need to correct the clocks of computers sharing a network. As some representative examples of networked DAQs, in [9], we can find a distributed DAQ system aimed at structural monitoring of constructions. Due to the large scale of a typical setup (hundreds of meters), that work proposes wireless radio communications between the sensing nodes, which synchronize their internal clocks following the Flooding Time Synchronization Protocol (FTSP) algorithm [10] and achieving a worst-case synchronization of 1 ms. Such time errors are more than acceptable for studying vibrations of large mechanical structures whose fundamental frequencies are within the range of a few Hz. In [11], wireless communications are also used to monitor structures, with analog signals being acquired at a rate of 50 S/s. Regarding cutting-edge experiments in physics, synchronization across sensors distributed all around the planet must often be ensured.

An example of such a system is the global network of optical magnetometers reported in [12], where 16-bit analog signals are sampled at a maximum rate of 1 kS/s in stations that can be at any location on the planet. As is usual in these cases, the pulse-per-second (PPS) signal from a Global Positioning System (GPS) receiver [7] is employed to correct the internal counters of a microcontroller to achieve a global synchronization better than ±200 ns. In the present work, we have not yet introduced the requirement of a GPS PPS signal, since the requisites of typical mechanical tests are not so demanding, but this could be done by modifying the firmware. See [5] for a more comprehensive review of other custom DAQ systems.

**Table 1:** Comparison of key features among other commercial and non-commercial USB DAQ devices, including their analog inputs (AI), digital inputs and outputs (DIO) and counter/timers ports (C/T).

DAQ Model	Key Features
Advantech USB-4702-AE	12-bit 10 kS/s ± 10 V 8 AI, 0–5 V 2 AO, 8 DI, 8 DO, 1 C/T 32-bit
Data Translation DT9812-10V	12-bit 100 kS/s 8 AI, 2 AO, 16 DIO, 1 C/T
HYTEK iUSBDAQ U120816	12-bit 13 kS/s 0–4.096 V 8 AI, 16 DIO, 1 C/T 16-bit
LabJack U12	12-bit 1.2 kS/s ± 10 V 8 AI, 2 AO, 20 DIO, 1 C/T 32-bit
MC USB-1208FS-Plus	12-bit 50 kS/s ± 10 V 8 AI, 16 DIO 0–5 V, 1 C/T 32-bit
NI USB-6008	12-bit 10 kS/s ± 10 V 8 AI, 2 AO 0–5 V, 12 DIO, 1 C/T 5MHz
Phidgets 8/8/8	12-bit 1 kS/s 0–5 V 8 AI, 16 DIO
PhidgetEncoder HS	4 DIO, 4 C/T (max. $10^{6}$ pulse/s)
SimpleDAQ [5]	12-bit 0–3.3 V 10 AI, 8 DIO
This work	16-bit 50 kS/s ± 10 V 8-32 AI, 8 DIO, 2-8 C/T (max. $4\times 10^{6}$ pulse/s)

To provide a comparison between the proposed modular platform and a representative selection of other similar off-the-shelf and non-commercial USB DAQ devices, we have summarized their key features in Table 1. The cost of the selected devices is the range of $100–400. As can be seen in the table, the proposed DAQ has the potential to offer a better SNR in analog signal acquisition due to its superior resolution. In addition, only two other devices included in this comparison can offer a sample rate of 50 kS/s or above. Regarding quadrature decoder or counter inputs, only the application-specific PhidgetEncoder board offers a similar number of high-speed counters, though with a more limited maximum speed.

The rest of this article is structured as follows. Section 2 gives a detailed description of our platform, including its hardware and firmware. Next, different tests and experimental validations are described in Section 3 and finally some conclusions are outlined in Section 4.

## 2. Proposed Modular Architecture

The DAQ proposed in this work belongs to the category of specialized modular designs, as mentioned above. The main objectives that have directed our design decisions are:

- Sampling frequency: At least 20 kS/s for 16-bit analog inputs and quadrature decoder counters. This rate should be sufficient for most DAQ tasks in mechanical engineering, industry process control, robotics, *etc*. These requisites imply a minimum communications bandwidth of $BW_{min}=1.28\;\text{MB/s}$ in order to stream 32 analog channels at 20 kS/s.
- Portability: The system should be easily attached to any computer running Microsoft Windows, GNU/Linux or Mac OS.
- Low cost, but without sacrificing the accuracy and performance attainable with other off-the-shelf commercial DAQ models.

**Figure 1:**
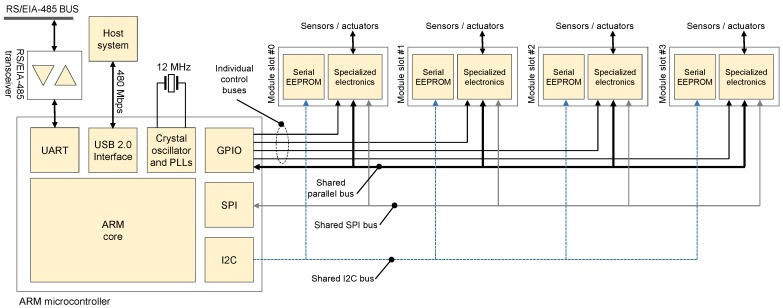
Simplified block diagram of our proposal DAQ platform. The main microcontroller communicates with the host computer via a high-speed USB 2.0 link while grabbing or sending data to the diverse modules via a number of specialized buses.

For that purpose, a custom set of buses has been designed for connecting a central microcontroller with a number of modules, different or not, installed in the four slots of the base board. Each of those modules is in charge of handling specialized inputs or outputs, as sketched in Figure 1 and further explained in the following.

### 2.1. Hardware Design

**Figure 2:**
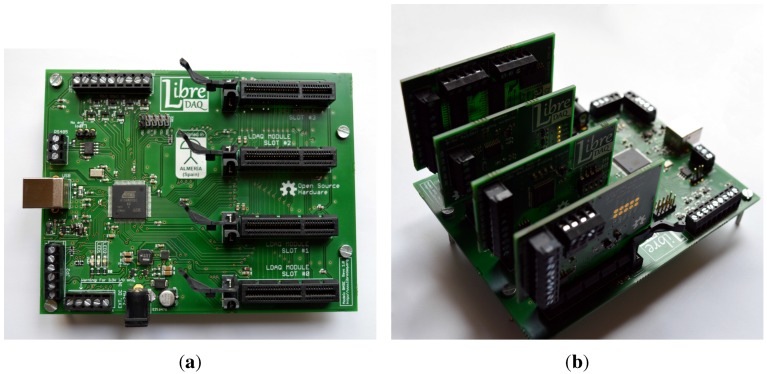
A prototype of the proposed system. (**a**) The base controller board, with the four empty slots at the right-hand side; (**b**) Base board with all its four slots populated with DAQ modules.

In the following, we provide details about some important design decisions that have been taken during the development of the custom DAQ. See Figure 1 for an overview of the different subsystems. A picture of our latest prototype is shown in Figure 2.

#### 2.1.1. Communications

An initial decision of paramount importance is whether the DAQ system would be installed inside or outside a computer. Such a decision determines the available communication buses, e.g., the use of PCI-Express if placed inside the computer, *versus* USB or Ethernet otherwise. Both options have pros and cons. Higher data rates are attainable with the internal PCI-Express 1.0 bus (typically 250 MB/s) in comparison to external setups, such as as High-Speed (HS) USB 2.0 (maximum 480 Mb/s or 60 MB/s), Full-Speed (FS) USB 2.0 (maximum 12 Mb/s or 1.5 MB/s) or 100BASE-TX Ethernet (maximum 100 Mb/s or 12.5 MB/s). Following our design criterion of achieving a portable DAQ system, suitable to be easily installed in any computer with different operating systems, we quickly discarded the use of internal buses. Therefore, the two natural options are the most popular external buses, *i.e*., USB and Ethernet. Our choice was USB due not only to its omnipresence in all kinds of computers, laptops and hand-held, as well as embedded systems, but also to the reduced cost of hardware requirements for its physical layer. Regarding the suitability of FS USB, our design criterion of $BW_{min}=1.28\;\text{MB/s}$ is too close to its maximum theoretical limit of 1.5 MB/s. In practice, sustainable rates of 1 MB/s constitute a more realistic maximum limit for FS USB. Therefore, to avoid the USB link from becoming a bottleneck, we chose to use HS USB 2.0 as the main communication link between our DAQ board and the host system. Notice in Figure 1 that an alternative communication link for RS/EIA-485 networking is provided via an Universal Asynchronous Receiver-Transmitter (UART) for the purpose of message exchanges across multiple instances of the proposed board. This feature allows building up a distributed DAQ to monitor, for example, an industrial plant, a robot or a vehicle. At present, the usage of this link is mainly devoted to the interchange of messages for synchronizing the internal clocks of all boards in a network, such that all timestamps have a consistent time reference.

#### 2.1.2. Microcontroller

Given the low-cost goal of our project, we considered the use of some of the most popular eight-bit microcontroller families, such as Microchip PIC or Atmel AVR, *i.e*., those employed in the popular BASIC Stamp and Arduino^(TM)^ boards, respectively. However, after carefully comparing their performance and price to 32-bit microcontrollers based on ARM cores, the latter became an obvious preferred choice due to their increased Million Instructions Per Second (MIPS) and higher clock frequencies. After these considerations and given the communication requirements discussed above (support for high-speed USB 2.0), we chose the 32-bit Atmel SAM3U ARM Cortex-M3 as the central microcontroller of our prototypes. A crystal of 12 MHz was used as the main clock source, which, after firmware-based configuration of the internal PLLs, became a system main clock of 96 MHz.

#### 2.1.3. Modules Connector

The choice for the connector between DAQ modules and the main board was made considering space economy, availability from different suppliers and robustness against real-world conditions. Our decision was to use standardized PCI-Express female connectors for the four slots in the main board, as shown in Figure 2a.

#### 2.1.4. Buses

**Figure 3:**
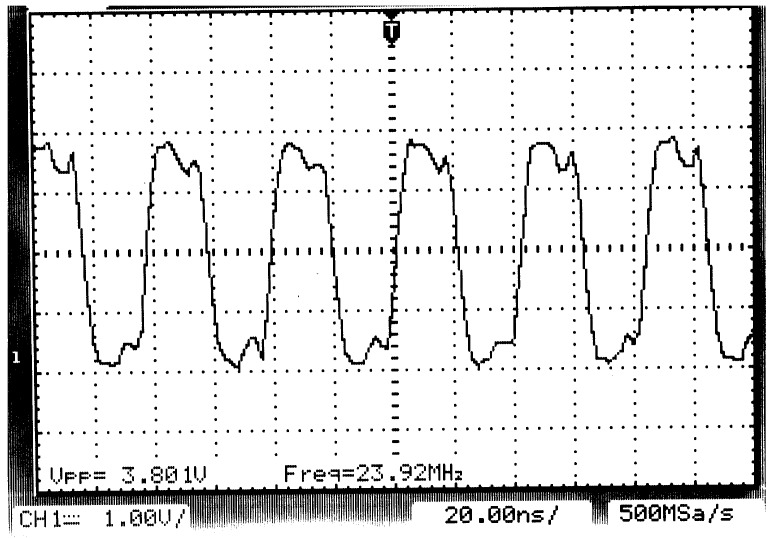
Oscilloscope image of the shared clock waveform as received into each DAQ module. The moderate overshoot and edge slopes contribute to a reduction of electromagnetic interference (EMI) due to radiation.

As can be seen in Figure 1, the central microcontroller communicates with each DAQ module by means of several separate buses:

- Shared lines: These include a bidirectional 8-bit data bus for use in chips supporting parallel access, and a shared configurable system clock for use inside each DAQ module. At present, this clock line is driven by a 3.3 V, 24 MHz signal internally generated in one of the microcontroller programmable clock sources. A small serial chip resistor has been added to this line to prevent excessive over-shoot (refer to Section 3.1 and Figure 3).
- Module-specific lines: A set of four independent lines have been placed between the microcontroller and each module to allow easy and safe access to module hardware signals even in the case of preemptive interruptions that may interfere with the execution of internal communication protocols for one module to give the control to another one.
- Serial Peripheral Interface (SPI) bus: For communicating with devices that support this fast serial protocol. The additional signals associated with this bus (typically, at least one chip-select line) are implemented via the module-specific signal bus.
- Plug-and-play Inter-Integrated Circuit (I^2^C) bus: The purpose of this bus is the reading (and programming) of a small, inexpensive serial Electrically Erasable Programmable Read-Only Memory (EEPROM) memory attached to every DAQ module. This memory contains a self-description of the “services” (analog inputs or outputs, *etc*.) offered by the board, as well as an unique numerical identifier that allows the selection of the proper driver module in the base board firmware for communicating with the module. Normally, this memory is read only once upon system start-up.

It should be noted that there is no global sample signal to mark the sample-and-hold of analog inputs in different modules. However, when simultaneous sampling is required, our firmware is designed for those signals to be generated in successive system clock cycles. Given the execution model of the ARM core and the system clock of 96 MHz, the maximum delay between the sample mark of different modules is 31.25 ns, which is negligible for the application fields at hand.

The microcontroller pins that are not devoted to these buses are exposed as additional external inputs and outputs. Careful selection of pin assignment for the buses results in the availability of free pins that can be configured to work as 3.3-V or 5-V General Purpose Input/Output (GPIO), programmable pulse-width-modulator (PWM) outputs or as an additional SPI bus for connecting digital sensors. As will be discussed in Section 2.2, services available at any of the four DAQ modules will be indexed from the computer software by slot number (from 0 to 3), while services exposed directly through these microcontroller peripherals use the special denomination of being embedded and can be selected by the special slot index -1 (or 0xff in two’s complement binary representation with one byte).

#### 2.1.5. DAQ Modules

The modular architecture of our design allows a great flexibility in the future development of DAQ modules as required by specialized applications. It should be recalled that each module must hold the appropriate electronics to adapt the interface of the chips of interest to the buses described above. In some cases, almost no additional components are required, but we found that a frequent problem is the need to convert inputs and outputs between the 3.3-V Complementary Metal-Oxide-Semiconductor (CMOS) logic levels employed by current ARM microcontrollers and another level, typically Transistor-transistor logic (TTL) or cmos 5.0-V levels.

In the present work, we describe experiments with two such modules:

- Analog inputs: This module, built around an Analog Devices AD7606 chip, features eight simultaneous sampling ADC channels with 16-bit resolution, parallel bus output and a maximum sampling frequency of 200 kS/s; although, due to firmware limitations, our prototype is capable of a maximum of 100 kS/s. The bipolar voltage input range is ±10V.
- Quadrature encoder inputs: Four 32-bit decoders (implemented with Avago’s HCTL-20XX chips), with the capability of simultaneous reading. The common system clock of 24 MHz is used as input to the decoder logic circuitry, which, due to the existence of a digital noise-suppressing stage on each input, leads to a nominal maximum of 4,000,000 input pulses per second or 1,000,000 full quadrature cycles per second. This limit is remarkably above most existing commercial solutions.

### 2.2. Software

The developed software can be clearly split into the firmware running on the ARM microcontroller, and the user-side Application Programming Interface (API) in the host computer. Next, we briefly describe their most notable features. Part of the software has been released online [13].

#### 2.2.1. Firmware

The firmware has been developed in C language, compiled with GNU GCC as x86-ARM cross-compiler and debugged with Atmel Studio using the Joint Test Action Group (JTAG) interface available in the microcontroller. The main building blocks for the firmware have been taken from the Atmel Software Framework (ASF) libraries [14].

We can summarize the tasks programmed into the firmware as follows. At start-up, all subsystems are initialized: system clock, interruptions, the USB stack, GPIO pins and peripherals for the DAQ buses, *etc*. Then, the main infinite loop just checks whether new commands have arrived from the host computer via USB (the reception is interrupt driven) and dispatches them according to a set of arbitrarily-defined numeric operation codes (“OPCODES”). Examples of such commands are: start the conversion of analog inputs for Slot 1 at 25 kS/s, stop all conversions, change the embedded PWM generator duty cycle to 25%, *etc*. The binary representation of such commands can be checked out in the online source code; refer to the header file ldaq_frames.h in [13].

In order to support an accurate timing of the requested tasks, a minimal, custom implementation of a round-robin scheduler has been developed. At present, the system tick frequency that drives this scheduler defaults to 50 kHz, a frequency that also defines the resolution of the real-time high-resolution 32-bit clock of the system, used for timestamping every single piece of data acquired from any sensor, *i.e*., observations have a temporal resolution of 1/50 kHz = 20 μs. Our claim of all sensors to be accurately sampled within ∼1 μs corresponds to the maximum duration imposed on the code executed for each DAQ module during one scheduler iteration.

A word is needed regarding the modifications that have been made to the standard Atmel ASF USB libraries. As a means of supporting atomic operations, ASF USB stack masks the global interrupt flag, a widespread and common approach in embedded systems programming. However, we observed that this practice led to unacceptable, unpredicted delays in time-based scheduler interruptions that mark the instant of sampling for DAQ modules. In other words, they introduced a significant jitter in the signal-sampling clock, in effect distorting the acquired signals. Our solution consists of exploiting the preemptive interrupt system of the ARM processor by carefully changing the interrupt priority levels of each subsystem. Therefore, by setting the maximum priority to time-based interrupt and an intermediary priority to USB communication events, the system is capable of robust USB communications while performing low-jitter sampling, as shown with a spectral analysis in Section 3.1.

#### 2.2.2. Host-Side API

A C++ API has been developed for interfacing our DAQ device from any operative system that supports USB-based serial ports. The main interface is the C++ class libredaq::Device (see [13]), which can be used to connect to a specific serial port and offers methods to start and stop the grabbing of different DAQ tasks. At present, the only reading-back mechanism is via callback functors provided by the users. Incoming USB data streams are processed in a detached thread in a way that is transparent to the user. Refer to the online source code for examples of usage. A Graphical User Interface (GUI) application is also under development, but it is not relevant for the experiments presented in this work.

## 3. Experiments and Discussion

This section describes some of the tests that our prototype has undergone to validate its suitability. Experiments have been separated into four groups. First, we summarize some of the low-level tests aimed at verifying the different subsystems of the modular DAQ. Next, the synchronization accuracy of signals from different DAQ modules is demonstrated and contrasted with that of a commercial DAQ. The third experiment consists of a demonstration of the kind of datasets that can be grabbed in the mechanical system. Finally, we provide results for impact-test experiments, a highly-demanding experiment in the specific feature our DAQ was designed to meet, *i.e*., accurate synchronization of heterogeneous, quickly-changing signals.

### 3.1. Low-Level Testing

Firstly, our prototype underwent a series of standard electrical checks to ensure the proper working of the most basic subsystems: power input, voltage regulators, crystal oscillator, *etc*. Next, increasingly complex firmware programs were tested to check the different ARM peripherals and the electrical connections in the boards. As an illustrative example, Figure 3 shows the output of the programmable clock generator used as a reference clock in the shared parallel bus (see Figure 1). Due to the relatively long distance that this signal must travel and its tree-like distribution pattern from the core microcontroller toward each module slot, a series resistor was placed near the clock driver to reduce a number of undesirable effects, such as open-line reflections and interference emissions.

**Figure 4:**
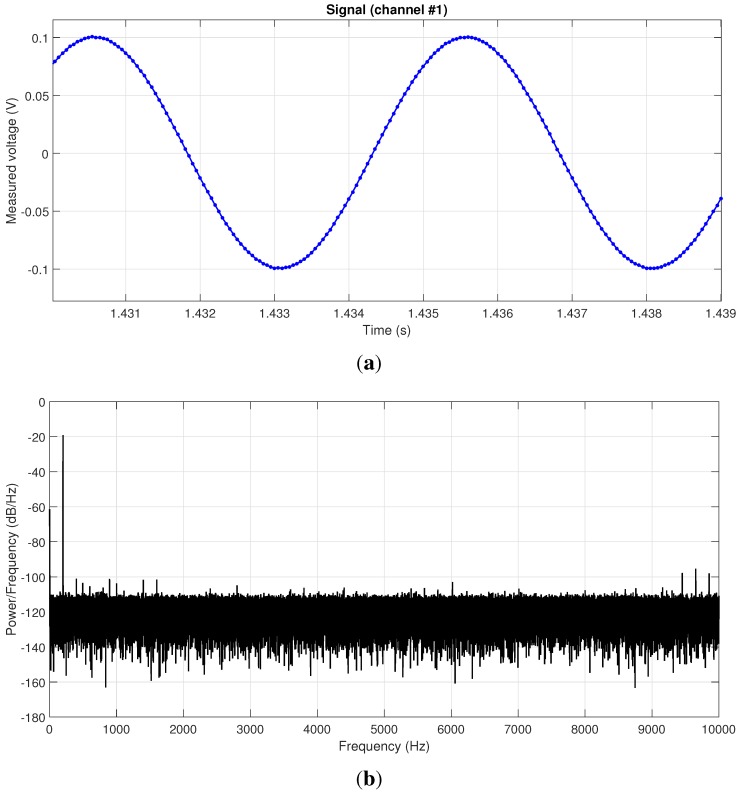
The results for a test consisting of feeding a 200 Hz sinusoidal signal into a 16-bit ADC DAQ module, are shown in (**a**) and (**b**) in the time and frequency domains (with Hanning windowing), respectively.

Another key test was related to the validation of analog signal acquisition. A sinusoidal signal of 200 Hz and 200 mVpp was generated with an Agilent 33220a signal generator, then acquired with the entire pipeline of hardware ADC, firmware on the ARM microcontroller and recorded in a desktop computer with the public user API. The result is shown in the time domain in Figure 4a. In order to verify the spectral quality of the acquired signal and discard artifacts, such that those caused by jitter are in the sampling frequency (refer to Section 2.2), we also computed the power spectral density of the signal (using a Hanning window). The result, in Figure 4b, demonstrates an excellent performance, with a very clean peak at 200 Hz, an average of a 90-dB Signal-to-Noise Ratio (SNR), with the theoretical prediction for an *N*-bit ADC and a sinusoidal input being 6.02N+1.76dB=98.08dB.

Finally, the limits of the USB communication channel were explored by sending a continuous stream of data at the maximum attainable speed allowed by the microcontroller firmware. We found that, despite the instantaneous theoretical limit of 480 Mbps (60 MB/s) allowed by HS USB, the chosen microcontroller could sustain a constant rate of only ∼4 MB/s, which, however, is far above the requirements of our DAQ system and superior to the limit of ∼1 MB/s obtained with FS USB.

### 3.2. Interchannel Synchronization

In this experiment, we demonstrated the accurate synchronization claimed by our design between heterogeneous signals, namely an analog input and a digital quadrature channel. Here, the experimental setup included an optical quadrature encoder, whose shaft was moved manually at a non-constant speed. We investigated the accuracy of the synchronization attainable by our custom board in comparison to a commercial device, a National Instrument (NI) USB-6210, by reading the encoder quadrature channels (A+ and B+) simultaneously through both devices’ quadrature decoders. Moreover, the A+ channel was also recorded by both devices as an analog signal. By doing so, the timing of quadrature edges in the analog signals could be compared with increments at the output of quadrature decoder counters. The sampling rate was 25 kS/s for both analog and encoder counters in our device, and 5 kS/s in NI USB-6210 under LabVIEW. Following the recommendations by the technical service of National Instruments in response to the inquiries by the authors concerning how to achieve the most accurate time stamping in LabVIEW, we employed a high resolution relative seconds block and saved datasets in the vendor-specific Technical Data Management Streaming (TDMS) file format.

**Figure 5:**
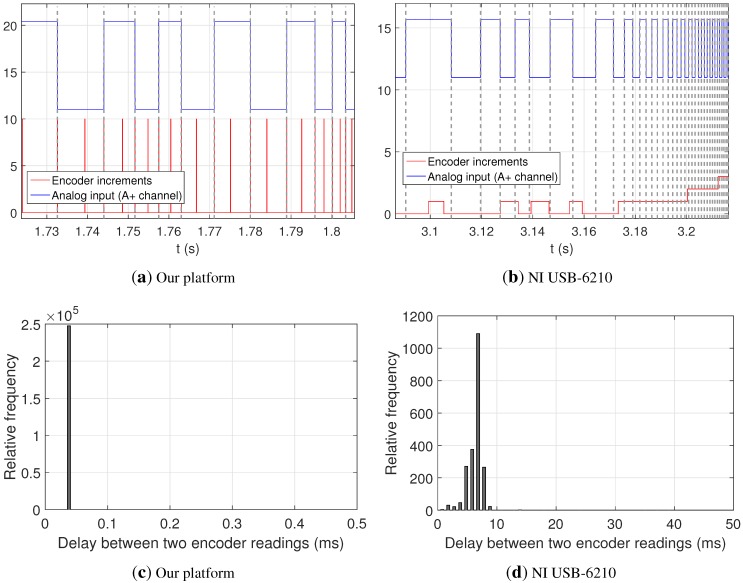
One channel (A+) from a quadrature encoder as an analog signal (blue) and increments in quadrature decoder counters (red) for both, our design (**a**) and a National Instrument USB-6210 board (**b**); For visual reference, we added vertical dashed lines marking the rising and falling edges of quadrature cycles. Vertical axes are not to scale; (**c**,**d**) Histograms of delays between the registration of counter values by each device. Refer to text for further details.

Ideally, we would expect counters to increase immediately (actually, a few nanoseconds afterward, depending on the frequency of the underlying digital logic clock, 125 ns for our design) after each quadrature rising and falling edge, due to the use of so-called “×4” decoders, which increase their count four times with each full quadrature cycle. The experiments showed that counter changes in our device are precisely aligned (within one sample period, τ=1/15kS/s≈66.7μs) with those edges, as shown in Figure 5a, where vertical dashed lines have been added for visual reference at the edges of each quadrature cycle. Notice how rising, as well as falling edges coincide with increments in the counter, while the other two increments per cycle correspond to the edges of the B+ channel, not shown in the figure. Such an accurate synchronization of events is clearly not present for the dataset captured with the commercial DAQ board, as can be seen by visual inspection of Figure 5b. To make a quantitative comparison of the regularity of counter readings by both platforms, we also computed the delay between consecutive readings. Ideally, these delays should match the inverse of the nominal sampling frequency for each device, that is, 1/25kS/s=40μs and 1/5kS/s=2.5ms for our platform and NI USB-6210, respectively. However, it can be seen in Figure 5c,d that an accurate sampling of encoder counters is achieved only with our device.

### 3.3. Mechanism Instrumentation

**Figure 6:**
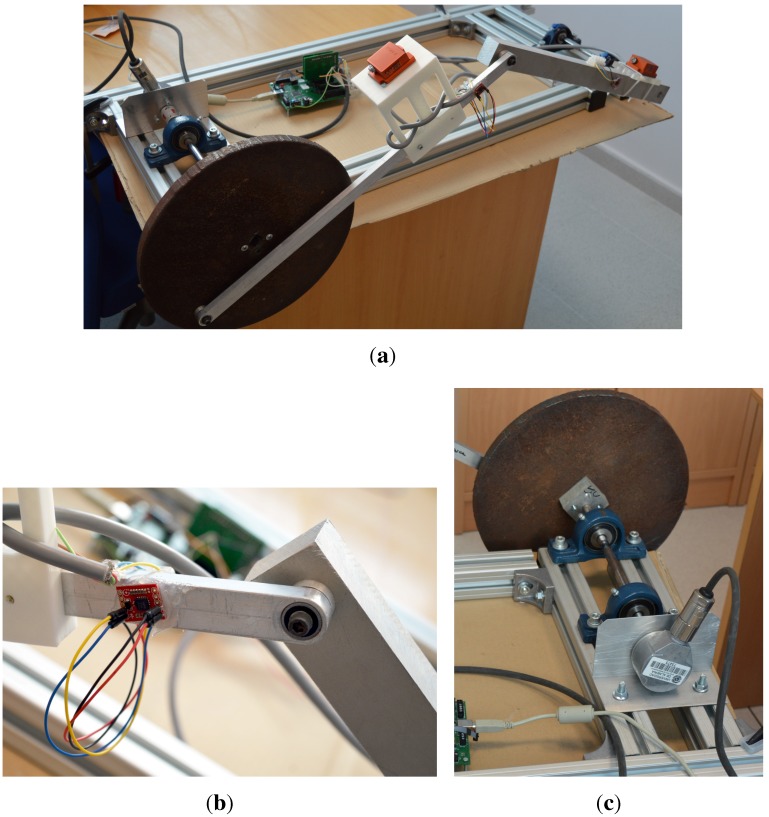
(**a**) Four-bar mechanism testbed; (**b**) Detail of the gyroscope mounted in the connecting rod; (**c**) Detail of the crank and optical quadrature encoder assembly.

An initial practical evaluation of the proposed DAQ system was the acquisition of a multi-sensory dataset for a four-bar mechanism used as a testbed for state estimators, shown in Figure 6 and already described in previous works [15].

**Figure 7:**
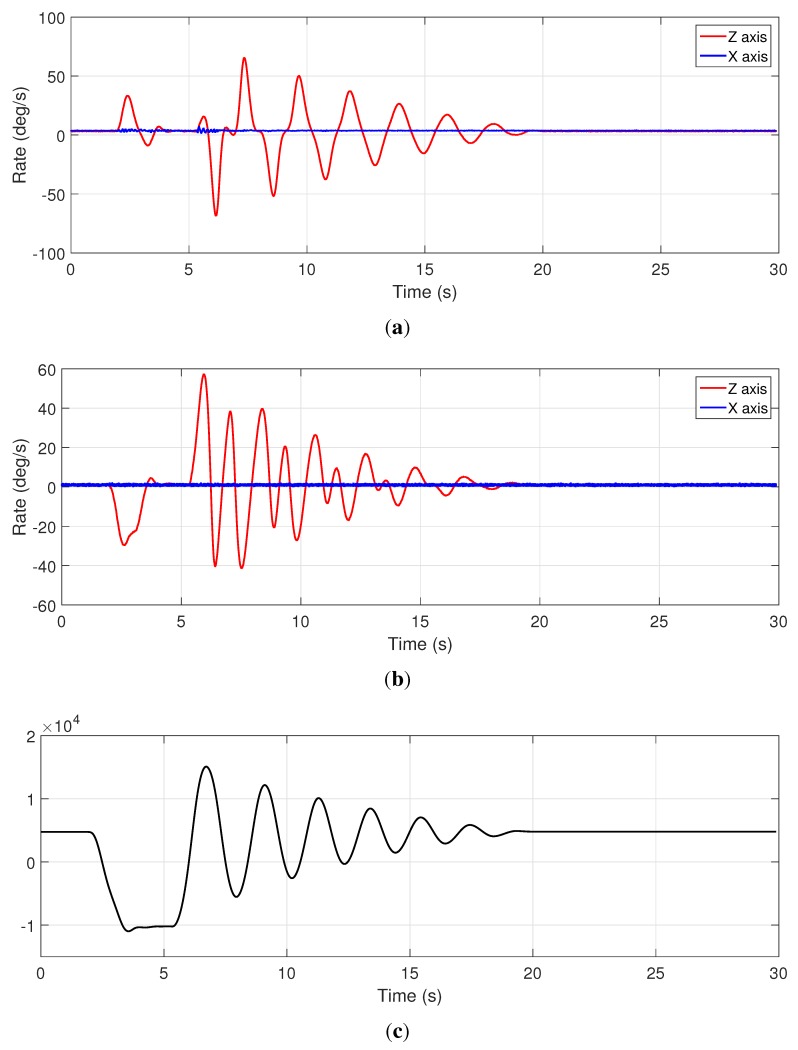
Measurements for the mechanism tested with the proposed DAQ system. Gyroscope readings for the connecting and rocker rods are shown in (**a**,**b**), respectively; Since the mechanism is planar, gyroscopes aligned with the *Z* axis provide the most significant measurements; Incremental encoder data is shown in (**c**), in pulse units (the encoder generates $40\times 10^{3}$ pulses per revolution).

In short, the mechanism consists of a four-bar linkage comprising (i) a high-inertia disk made of iron operating as crank link, whose axle is connected to an optical quadrature encoder; (ii) a soft bar built in aluminum acting as connecting rod in which the first gyroscope is placed and (iii), a solid rod made of aluminum as rocker link, incorporating another identical gyroscope. In comparison to previous works, we have replaced two professional-grade XSens MTi Inertial Measurement Unit (IMU) with low-cost dual analog gyroscopes (LPY503AL). Then, the proposed DAQ is used to record the signals from the two gyroscopes in the connecting and rocker rods of the mechanism simultaneously to the orientation measured by the optical quadrature encoder. These datasets can be used as inputs to estimation algorithms in mechanical engineering, and it will be future study as a continuation of former works in [16,17]. Refer to Figure 7 for a description of the acquired dataset, which shows an excellent synchronization of the different sensors, as can be visually assessed in a qualitatively way. During this experiment, the mechanism was manually moved out of its equilibrium configuration (around t = 2 s), then it is dropped (around t = 5 s) under the unique effect of its own weight and it starts oscillating until it stops due to friction. The next experiment is designed to test our device under much more rigorous conditions.

### 3.4. Impact Tests

Impacts represent events where changes in the dynamical state of the involved bodies happen in a very short time, hence becoming an excellent challenge to our DAQ aimed at measuring heterogeneous signals with a common reference clock.

**Figure 8:**
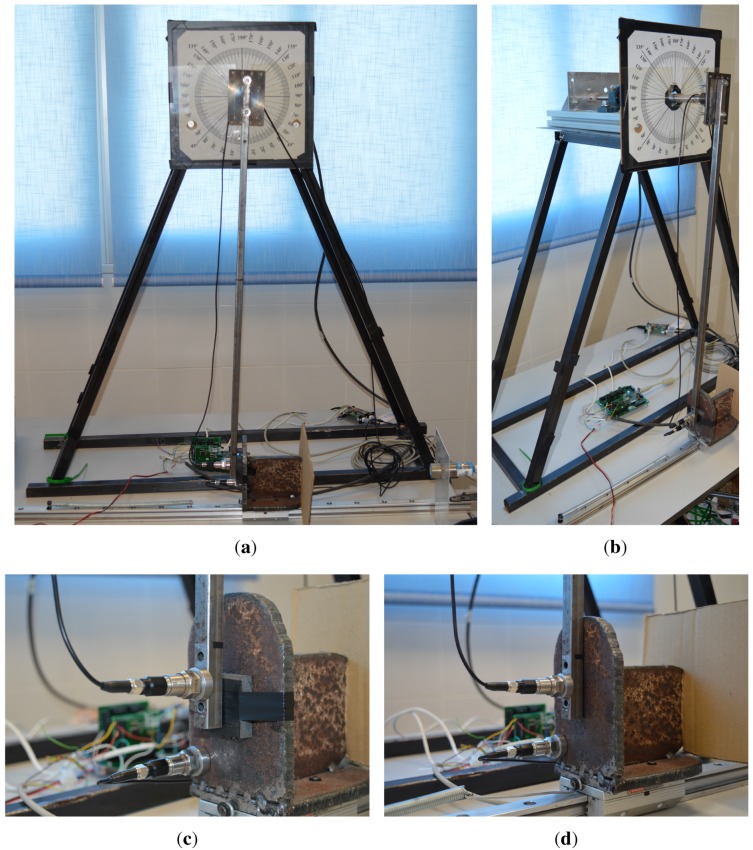
(**a**,**b**) Views of the impact testbed used in these experiments. The rigid rod is standing still in vertical position here, touching the mobile mass on a linear bearing at the bottom; (**c**) A close-up view of the two accelerometers, on the rod and the mass, with foam installed at the impact point; (**d**) The same view for the tests without protective foam.

The impact testbed used is shown in Figure 8. It consists of a pendulum mechanism and a “dummy”. A horizontal axle, attached to an optical quadrature encoder, is firmly joined to a slender rod, which in the pictures is shown resting in its vertical (equilibrium) position. At the bottom, we can find a horizontal sliding mass (a “dummy” body), which is kept in place by means of a linear spring. Impact tests are performed by raising the rod (in the clockwise direction, as seen in the pictures), then dropping it in such a way that it hits the mass at the bottom. Depending on the mass of the rod and the “dummy”, their mechanical characteristics, and the covering material placed in the contact area, different phenomena can be observed at the instant of collision. The testbed was already described in previous works [18], while analyzing safety issues in robot arm-human collisions. For more details, please see that work on this testbed.

**Figure 9:**
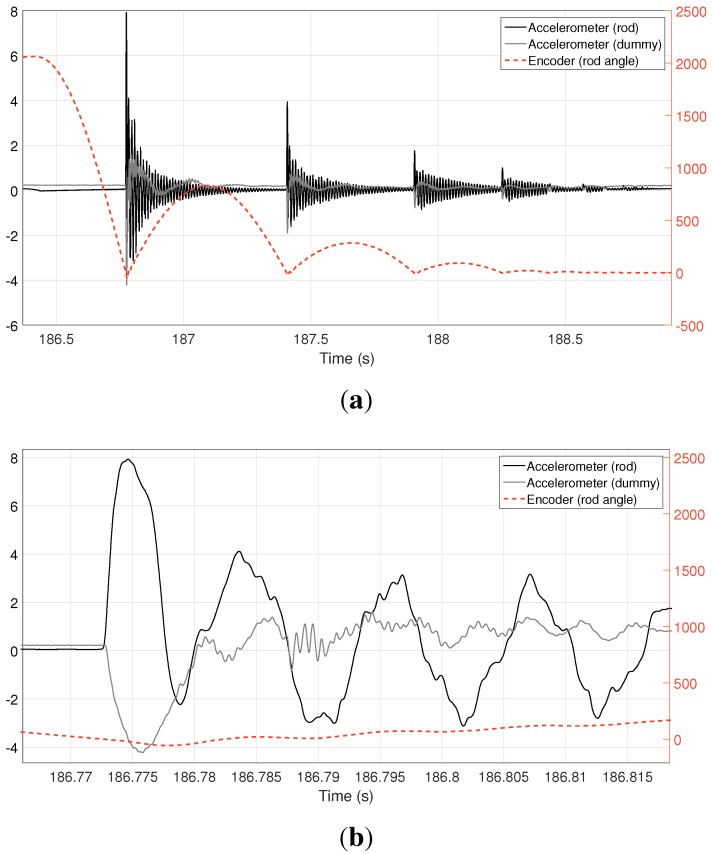
(**a**) Overview and (**b**) close-up of the collision test. Left-hand vertical axis represents voltage for accelerometer readings (after signal conditioning), whereas the right-hand axis stands for pulse counts for the encoder attached to the rod axle.

The sensors used in this experiment were two piezoelectric accelerometers, attached to the rod and to the “dummy” mobile mass at the bottom, and the quadrature encoder of the rod axle. Several tests were conducted using different material covers at the impact point, including a thick foam cover and no covering at all. Figure 9 shows an overview of all of the acquired signals for the case of using the foam covering. The first impact is clearly seen at t = 186.77 s, with at least four more collisions caused by rebounds of the rod; refer to the rod angle, measured by the encoder and plotted as a dashed, red line. A close-up of the signals upon the first impact is shown in Figure 9b, where it is obvious that a delay(∼2–5 ms) exists between the impact, as detected by the accelerometers, and the detection of any significant effect at the other end of the rod (see the encoder signal), probably caused by the elastic deflection of the rod due to its kinematic energy. Another expected outcome is the generation of a complex vibration pattern in both the rod and the dummy mass.

**Figure 10:**
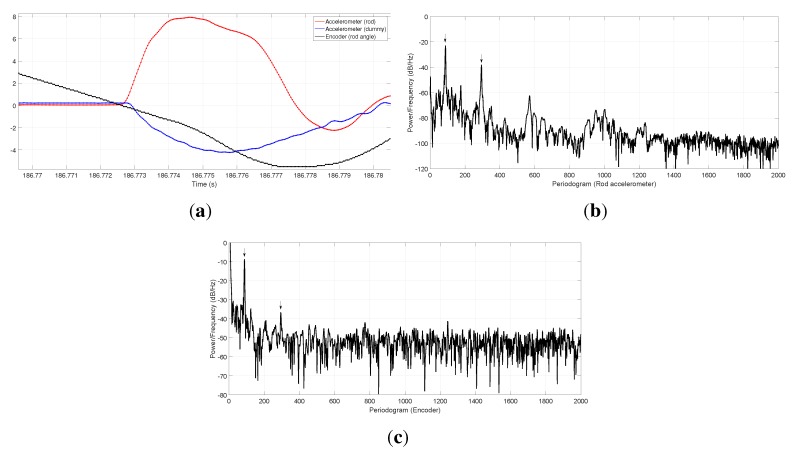
(**a**) Signals at the instant of impact, with a more detailed time scale; (**b**) Spectral density of the rod accelerometer during one rod oscillation period after the impact; and (**c**) the same spectral density but computed from the optical encoder data.

A notable observation allowed by the high sampling rate of our system is the experimental determination of the time required by the impact to reach, through the foam cover, the accelerometer in the “dummy” mass. Another close-up view of the instant of impact with even higher temporal resolution, shown in Figure 10a, reveals a delay between the responses of both accelerometers. The rod seems to start noticing the impact at t = 186.772300 s, whereas the dummy response starts at t = 186.772820 s, giving a measured delay of ∼520 μs. Regarding the encoder data at the other end of the rod, the approximate constant velocity that can be appreciated during the last few milliseconds immediately before the collision is affected only about 2.74 ms after the impact, which could be explained again by the body elastic properties.

In addition to these time domain tests, the vibration spectrum of the rod after the impact has been determined by means of the power spectral density (PSD) of the signals from the rod accelerometer and the rod optical encoder, leading to the results shown in Figure 10b,c, respectively. At least two clear fundamental frequencies are visible in both spectra, the first at 87.4 Hz in the accelerometer data (87.25 Hz for the encoder) and the second at 293.9 Hz in the accelerometer data (293.5 Hz for the encoder). These results are an important success, since they demonstrate the consistent sampling of heterogeneous signal types, *i.e*., analog voltage values from the accelerometers and digital counters from quadrature decoders, the latter having a discrete nature (less resolution), hence a more reduced SNR than the former.

## 4. Conclusions

This work has proposed a custom hardware and software design for a low-cost, modular DAQ platform. The main features of the proposal are its modular design and the possibility of sampling heterogeneous data sources from a single microcontroller, which enables referencing all measurements to a consistent and precise time reference. This is the first work available in the technical literature on vibration analysis in which the system allows the spectral response of a vibration to be obtained from an optical encoder thanks to the accurate, high-rate sampling of heterogeneous data sources, such as quadrature signals.
